# Stakeholders' Attitude to Genetically Modified Foods and Medicine

**DOI:** 10.1155/2013/516742

**Published:** 2013-12-05

**Authors:** Latifah Amin, Jamaluddin Md Jahi, Abd Rahim Md Nor

**Affiliations:** ^1^Centre for General Studies, Universiti Kebangsaan Malaysia (UKM), 43600 Bangi, Selangor Darul Ehsan, Malaysia; ^2^Institute of Malay World and Civilization, Universiti Kebangsaan Malaysia (UKM), 43600 Bangi, Selangor Darul Ehsan, Malaysia; ^3^Faculty of Social Sciences & Humanities, Universiti Kebangsaan Malaysia (UKM), 43600 Bangi, Selangor Darul Ehsan, Malaysia

## Abstract

Public acceptance of genetically modified (GM) foods has to be adequately addressed in order for their potential economic and social benefits to be realized. The objective of this paper is to assess the attitude of the Malaysian public toward GM foods (GM soybean and GM palm oil) and GM medicine (GM insulin). A survey was carried out using self-constructed multidimensional instrument measuring attitudes towards GM products. The respondents (*n* = 1017) were stratified according to stakeholders' groups in the Klang Valley region. Results of the survey show that the overall attitude of the Malaysian stakeholders towards GM products was cautious. Although they acknowledged the presence of moderate perceived benefits associated with GM products surveyed and were moderately encouraging of them, they were also moderately concerned about the risks and moral aspects of the three GM products as well as moderately accepting the risks. Attitudes towards GM products among the stakeholders were found to vary not according to the type of all GM applications but rather depend on the intricate relationships between the attitudinal factors and the type of gene transfers involved. Analyses of variance showed significant differences in the six dimensions of attitude towards GM products across stakeholders' groups.

## 1. Introduction

There has been significant advancement in modern biotechnology worldwide in the past ten years. Current biotechnology products mostly focus on the commercialization of biopharmaceuticals [1] followed by genetically modified (GM) crops [2]. Following the approval of recombinant human insulin for marketing in the United States in 1982 [3], the pharmaceutical industry has since then grown rapidly and, by the year 2009, 200 biopharmaceuticals have been approved for marketing [1]. The first generation biopharmaceuticals in the 1980s and early 1990s were classified as “simple replacement proteins” while an increasing proportion of second generation or modern biopharmaceuticals have been engineered to tailor their therapeutic properties [1, 3]. According to James [2], GM crops are the fastest-adopted crop technology in the history of modern agriculture with an unprecedented 87-fold increase between 1996 and 2010. Four major GM crops, maize, soybean, cotton, and canola covered almost 150 million hectares in 2010 with 29 countries which represent 59 percent of the world population planting GM crops. James [2] was cautiously optimistic that GM crops can meet the 2015 Millennium Development Goals of food security and poverty alleviation.

In Malaysia, biotechnology has been identified as one of the five core technologies that will accelerate the country's transformation into a highly industrialized nation by 2020 [4]. Most of the biotechnology activities in Malaysia are still under R&D except for delayed ripening papaya which has been approved by the Genetic Modification Advisory Committee (GMAC) for contained field trial. Being an agriculture-based nation, the strength of biotechnology in Malaysia is in agricultural biotechnology which is envisaged as a potential powerful tool to ensure food security and to boost the country's economy. Under the National Biotechnology Policy [5], health-care related biotechnology, industrial biotechnology, and bioinformatics are also given priorities by the Malaysian Government. Although modern biotechnology products developed by Malaysian researchers have not been commercialized yet, genetically modified (GM) foods and medicine from other countries are slowly coming into the country. Several imported GM foods already officially available in the Malaysian market are glyphosate resistant soybean (GM soybean) for human consumption and four types of GM corns meant for human food and animal feed. On the other hand, at least 26 biopharmaceutical products of modern biotechnology techniques have already been registered with the Ministry of Health, Malaysia, (MOH) for use in this country. The import, export, and release of genetically modified organisms (GMOs) in Malaysia is governed by the Biosafety Act 2007, which has been passed by the Malaysian Parliament in July 2007 and many activities have been initiated by the Ministry of Natural Resources and Environment (NRE) to work towards implementation of the Act. However, many people are not aware of the existence of the Biosafety Act and its role in governing biotechnology related research and industries in Malaysia [6]. The Biosafety Act (2007) contains only the core provisions; the details of the regulations such as risk assessment and risk management procedures will be developed by the Department of Biosafety, NRE. The Act did not cover the labelling requirement for GM foods and products. The Ministry of Health, Malaysia, is still working on the terms for labelling in Malaysia meaning that GM foods and products are not yet subject to mandatory labelling in Malaysia.

The advancement in gene technology for the production of GM crops and biopharmaceuticals has been so rapid in the past fifteen years, making it the object of an intense and divisive debate worldwide. The acceptance of gene technology varies from country to country and across different applications of the technology [7]. Past surveys have indicated that the Americans were generally more positive towards gene technology than the Europeans [7–11]. Costa-Font et al. [10] showed the evidence that worldwide consumers were not very supportive of GM crops. Gaskell et al. [11] and Christoph et al. [12] confirmed that the Europeans were as reluctant as in an earlier survey in 2005 [9] to support GM food and GM crops as compared to GM medicines. In Asia, majority of studies reported that the support for GM foods and medicines were not very encouraging too [13–20] with the exception of the urban consumers in India who were more positive towards GM crop [21]. The difference in attitude was because people were more concerned about the health, environmental, social side effects, and uncertainties of GM foods than those of GM medicine [22, 23]. The supporters of GM products envisage their potential benefits while the opposition groups view them as risky and tampering with nature [24]. Sagar et al. [25] suggested that a major factor in the emergence of controversies surrounding biotechnology has been the neglect of the needs, interests, and concerns of the primary stakeholders—the commoners. Connor and Siegrist [26] have proven that survey studies using questionnaires mirror people's perceptions well. Therefore, public perceptions, understanding, and acceptance of genetically modified organisms (GMOs) and GM foods can both promote and hamper commercial introduction and adoption of new technologies [23, 27]. Public acceptance can be understood as the combined attitudes of individuals on certain issues, such as those arising from technological innovations [28]. An individual's attitude towards a new technology depends on a number of related factors such as his or her perception of its risks and benefits, socially communicated values, and trust in institutions representing these technologies. With respect to public perception of genetic modification, Kelley [29] proposed that attitudes to genetic engineering are determined by the perceived worth of potential benefits offered, knowledge of genetic engineering, and having a scientific world-view, discounted by the perceived risk (rational worries) and anxieties or fears (irrational worries) and various minor factors such as background variables. Other studies have concluded that the acceptability of biotechnology by the public is primarily driven by risks and benefits perception [9, 26, 30, 31] as well as moral considerations [26, 32, 33]. Gaskell et al. [9, 34, 35] used four dimensions of attitude: perceived use, risk, moral acceptability, and encouragement to model patterns of European public response to biotechnology and GM foods.

The studies of public attitudes towards biotechnology and GM foods have many similarities with risk perception studies. The psychometric approach suggests that the public do not perceive technological risk according to a single dimension related to predicted injuries or fatalities akin to a risk assessor's viewpoint but interpret risk as a multidimensional concept, concerned with broader qualitative attributes [26, 36]. Within this approach, multidimensional risk perception is invoked to explain the expert-lay disagreement that is ascribed to lay ignorance in the knowledge deficit model [37]. The key variables of risk perception research are the perceived magnitude of risk or dread, risk acceptance, and familiarity with the hazard while recently the benefit factor has gained much interest [38]. Sjöberg [39] has highlighted the importance of another dimension: “interference with nature” in risk perception studies on genetic engineering.

The objective of this paper is to assess and compare the attitudes of the Klang Valley stakeholders towards two GM foods: genetically modified (GM) soybean (involving the transfer of bacterial genes into soybean to make it resistant to herbicide), GM palm oil (involving the modification of oil palm genes to reduce its saturated fat content), and GM insulin (involving the transfer of human genes into bacteria). GM soybean and GM insulin are already available in the Malaysian market while GM palm oil is a high priority area of research in Malaysia.

## 2. Materials and Methods

### 2.1. Survey Data Collection

A survey was carried out between June 2004 and February 2005. The people in the Klang Valley region were chosen as the targeted population as it is the centre of the country's economic and social development (with numerous universities and R&D institutions, biotechnology related industries) as well as the respondents in this region meet the requirement of diverse background stated in the model.

In this study, the stakeholder-based survey approach recommended by Aerni [28] was adopted but a wider range of interest groups were included (Table 1). Since the respective populations of the stakeholders involved were unknown, the respondents were chosen using stratified purposive sampling technique as recommended by McGrew Jr. and Monroe [40]. This technique enabled comparisons among respondents from different stakeholder groups that might otherwise be underrepresented if random sampling is used. The respondents (*N* = 1017) were adults (age 18 years old and above) stratified according to various interest or stakeholder groups listed in Table 1. Taking into account that this study was quantitative, the minimum effective size required for each statistical analysis was considered. Comparison of attitude across stakeholders (13 groups) was to be carried out using ANOVA. In order to have medium effect size (*f* = 0.25) at *P* = 0.05 and *u* = 12, a sample of 22 subjects per group is required to obtain a power of 0.80 [41]. So each stakeholder group except for the general public was allocated a minimum sample size of 22 but the number was increased where possible to take into account that some questionnaires might be incomplete or when the population size was bigger. Taking into consideration the recommendation by Krejcie and Morgan [42], for any population size beyond 5,000 a sample size of 400 would be adequate but would be more confident with a sample of 500, so the general public was allocated 550 respondents.

The questionnaires were handed out personally to respondents by biotechnology graduate enumerators who were trained to be neutral on their stance of GM products. Before answering, the respondents were given an introduction to basic concepts and examples of GM foods and GM medicine. They were also exposed to the real scenario of GM products debate on the possible benefits and risks and regulation of GM products, and they were given the chance to enquire further. This approach was suggested by Kelley [29] to assess unsophisticated public attitude on complex issues like modern biotechnology. Sturgis et al. [43] have shown that the provision of information prior to the survey did not affect people's attitude to biotechnology. Using this approach, the respondents do not have to know anything about biotechnology concepts and developments in the past years. They were introduced to the basic concepts and examples of biotechnology applications. Then they only have to read the questions and respond to the particular, concrete proposals in them—a far easier task. This style works perfectly well for sophisticated respondents as well as unsophisticated respondents besides allowing the researchers to use sophisticated statistical multivariate procedures to discover whether the attitude responses are empirically sensible. By using a multiplicity of questions, measurement errors are reduced [29].

### 2.2. Instrument

The multidimensional instrument measuring attitudes towards GM foods and medicine used in this study was constructed based on earlier research [44]. The instrument incorporated six dimensions of attitude consisting of the four dimensions used by the Eurobarometer surveys [34, 35]: perceived benefits, perceived risks, moral concerns, and encouragement with two additional dimensions frequently used in risk perception studies: familiarity [45, 46] and risk acceptance [38]. The items listed in Table 2 were measured in 7-point Likert scales from the lowest level of agreement to the highest level.

### 2.3. Data Analysis

Initially, reliability (Cronbach's alpha) tests were carried out using SPSS version 12.0 to assess the consistency and unidimensionality of the constructs. Analyses of variance (ANOVAs) were also carried out using the same statistical package. Confirmatory factor analysis was carried out using Analysis of Moment Structure (AMOS) software version 19 with maximum likehood estimation to validate the measures.

### 2.4. Confirmatory Factor Analysis

A single step SEM analysis as proposed by Hair Jr et al. [47] was carried out to estimate the measurement model using AMOS version 5.0 software with maximum likehood function. The chi-square goodness of fit index was statistically significant (*χ* ^2^ = 608.2, df = 208, *P* < 0.001). Chi-square statistical significant test is not very useful in indicating the fit of the model in this study due to the large sample size [48]. Other fit indexes have been proposed by several researchers to evaluate the fit of a model. CMIN/DF value of 3 or less, NFI greater than 0.9 [49], NFI, CFI, GFI, and AGFI greater than 0.9, and RMSEA value of 0.05 or lower supported with narrow confidence interval [49, 50] are recommended to indicate a well-fitting model. The measurement model for attitude towards modern biotechnology application in this study was found to have a good fit with CMIN/DF = 2.76, CFI= 0.97, GFI= 0.95, NFI = 0.95, and RMSEA = 0.042 with 90% confidence level in the range of 0.038 and 0.046.

### 2.5. Reliability

Three types of reliabilities measured in this paper are the internal consistency (Cronbach's alpha), item reliability, and construct reliability. The Cronbach's alpha coefficients for majority of the constructs were considered good (above 0.70) (Table 2). The corrected item-total correlations for all items in each dimension were very good (correlation coefficients greater than 0.5) (Table 2). The construct reliability is represented by the composite reliabilities and the variance extracted. From Table 2, it can be seen that the composite reliabilities for all the constructs were above 0.7 and the variance extracted (AVE) were all above 0.50 indicating good construct reliability [47].

### 2.6. Validity

Two validity measures were tested in this paper. The convergent validity was assessed by the factor loadings and composite reliability [47]. The standardized loadings of all factors were greater than 0.7 and the composite reliabilities for all factors were also above 0.7 indicating good convergent validity (Table 2). The discriminant validity was assessed by comparing the square root of average variance extracted (AVE) for each construct with the correlation between the construct and other constructs [51].  Table 3 shows that the discriminant validity is acceptable as the square roots of AVE for each construct were greater than the correlations between the construct.

## 3. Results

Attitudes towards GM foods (GM soybean and GM palm oil) and GM insulin were analyzed based on six dimensions: familiarity, perceived benefits, perceived risks, risk acceptance, moral concerns, and encouragement.

### 3.1. Familiarity

The Klang Valley stakeholders claimed that they were not very familiar with the three GM products surveyed. The overall weighted averages of familiarity level of the three products were below the mid-point value of 4.0 (Table 4). Of the three products, GM soybean was perceived as the least familiar followed by GM insulin and GM palm oil. Analyses of variance were significant for familiarity of GM soybean (*F* = 4.26, *P* < 0.001), GM palm oil (*F* = 5.39, *P* < 0.001), and GM insulin (*F* = 5.22, *P* < 0.001) across stakeholders. The biology students scored the highest weighted average in terms of familiarity with the three GM products (the only group with two ratings above the mid-point value of 4.0) and post hoc tests showed that their rating of GM insulin differed significantly from majority of other stakeholders except for the producers (Table 4). Their level of familiarity with GM palm oil was found to be significantly higher than three religious scholars (the Islamic, the Buddhist, and Christian), the biologists, and policy makers while their rating of GM soybean differed significantly from the Christian scholars. The weighted averages for familiarity of the religious scholars towards the GM products were among the lowest. The familiarity level of the Islamic, Buddhist, and Christian scholars with GM palm oil and GM insulin was significantly lower than the biology students while the familiarity rating of GM soybean by the Christian scholars also differed with the biology students. On the other hand, the rating of the Hindu scholars was significantly lower than the Biologists. It is also rather worrying that the familiarity level of the scientists (biotechnologists and biologists) and the policy makers was also similar with the majority of other stakeholders where their ratings of GM insulin were found to be significantly lower than the biology students. The familiarity level of the biologists and policymakers towards GM palm oil was in the same category with the religious scholars where their ratings were also significantly lower than the biology students.

### 3.2. Perceived Benefit

The overall benefits of all three GM products were in the moderate range with overall weighted averages above the mid-point of 4.0 (Table 5). The Klang Valley stakeholders perceived GM palm oil as having the highest benefit followed by GM insulin and GM soybean. These findings tend to suggest that when a GM application was perceived as having clear benefit to consumers, the application is ranked as the most beneficial. Palm oil modified to reduce its saturated fat content was ranked as the most beneficial. Next, GM insulin, which also has clear benefit to consumers, was perceived as having more benefits compared to GM soybean. On the other hand, GM soybean which did not seem to present clear direct benefit to consumers was ranked as the least beneficial.

Analyses of variance were significant for the perceived benefits of GM soybean (*F* = 2.92, *P* < 0.01), GM palm oil (*F* = 4.78, *P* < 0.001), and GM insulin (*F* = 5.22, *P* < 0.001) across stakeholders. Post hoc analyses of the beneficial aspects of the three surveyed GM applications highlighted the significant difference in opinion of the biology students compared to the media and the general public (Table 5). Their perceived benefit of GM soybean, and GM palm oil was also significantly higher than the Christian scholars, additionally differed from the NGOs in their rating of GM soybean, and differed further from the Hindu scholars with respect to GM insulin. The producers and the policy makers perceived high benefits of GM palm oil compared to the media while the biotechnologist's opinion of GM insulin was found to be significantly higher than the media. On the other hand, the media perceived significantly lower benefits of the three GM products compared to the biology students. Their rating of the benefits of GM insulin was also significantly lower than the biotechnologists while their opinion of GM palm oil was additionally lower than the producers and the policy makers (Table 4).

### 3.3. Perceived Risk

Overall, the respondents perceived the risk aspects of the three surveyed GM products as moderate with overall weighted averages above the mid-point of 4.0 (Table 6). Among the three GM applications, GM soybean was regarded as the most risky (weighted average 4.78) followed by GM insulin (weighted average 4.52) and GM palm oil (weighted average 4.35). The media were the most critical compared to other stakeholders. They perceived the highest risk for all three surveyed GM applications (Table 6). Analyses of variance were significant for the perceived risks of GM soybean (*F* = 1.76, *P* < 0.05), GM palm oil (*F* = 1.93, *P* < 0.05), and GM insulin (*F* = 3.22, *P* < 0.001). Post hoc test showed the media's rating of GM insulin as significantly differed from the biologists, policy makers, and biology students (Table 6) but post hoc tests could not detect significant differences in the risk ratings of GM soybean and GM palm oil.

### 3.4. Risk Acceptance

The overall weighted averages for the three GM products surveyed were about the mid-point score of 4.0, indicating that the stakeholders perceived the acceptance of risks associated with those products as moderate (Table 7). The risks associated with GM palm oil were ranked as the most acceptable (weighted average 4.14) as it has clear benefit to consumers and does not involve inter- or intraspecies gene transfer. Genetically modified insulin, with clear benefits but because it involves interspecies gene transfer, its associated risks were less acceptable (weighted average 4.02) compared to GM palm oil. On the other hand, GM soybean which involves the transfer of bacterial genes into soybean, with no clear benefits to consumers, made its associated risk to the least acceptable (weighted average 3.88). Analyses of variances were significant for the risk acceptance of GM soybean (*F* = 3.14, *P* < 0.001), GM palm oil (*F* = 2.70, *P* < 0.01), and GM insulin (*F* = 3.88, *P* < 0.001) across stakeholders. The media was noticeably the most critical with the lowest rating for GM soybean and GM palm oil. Post hoc tests showed that their acceptance of risk for GM soybean, GM palm oil, and GM insulin were significantly different from the biology students and additionally differed with the Buddhist scholars with respect to the acceptance of risk related to the two GM foods (Table 6). Being the highest scorer for risk acceptance of the three GM products, the biology students' rating differed significantly from the media (Table 7). Their acceptance of the risk associated with GM palm oil and GM insulin further differed with the general public while their rating of GM insulin was also significantly higher than the NGOs, the Christian, and Hindu scholars. Among the religious scholars, the Buddhists were the most accepting of the risk related to GM soybean and GM palm oil. Post hoc tests affirmed their level of risk acceptance of GM soybean and GM palm oil as significantly higher than the media. The Hindu scholars were found to be the most sensitive with the use of human gene in bacteria for the production of insulin. Their rating was the lowest and post hoc test confirmed that their opinion differed significantly with the biology students.

### 3.5. Moral Concerns

When confronted with the moral aspects, the stakeholders perceived the three GM products as raising moderate moral concerns (overall weighted average about the mid-point value of 4.0, Table 8). Genetically modified palm oil was seen as raising the least concerns (weighted average 3.84) followed by GM insulin (weighted average 4.01) and GM soybean (weighted average 4.05).

The Buddhist and the Christian scholars considered the three GM products surveyed as raising high moral concerns in contrast with the producers who regarded them as of low moral concern (Table 8). The rest of the stakeholders perceived the moral aspects of the three GM applications as moderate. Analyses of variances were significant for moral concerns about GM soybean (*F* = 9.30, *P* < 0.001), and GM palm oil (*F* = 8.96, *P* < 0.001), and GM insulin (*F* = 10.67, *P* < 0.001). Post hoc tests confirmed that the Buddhist and Christian scholars significantly perceived the moral aspects of the GM applications surveyed as higher than the majority of stakeholders except for the media and Hindu scholars (Table 8). The Hindu scholars were the next highest in rating the moral aspects of the three GM products. Post hoc tests again showed that their opinion of all three GM products was significantly different compared to the producers. On the other hand, the producers indicated low moral concern for all three surveyed products compared to six other stakeholders, the NGOs, the media, the Buddhist scholars, the Christian scholars, the Hindu scholars, and the general public. Their rating of GM insulin further differed from the biologists and politicians as well (Table 8).

### 3.6. Encouragement

Overall, the Klang Valley stakeholders were the most supportive of GM palm oil (weighted average 4.86) followed by GM insulin (weighted average 4.44) and GM soybean (weighted average 4.29, Table 9). Five groups of stakeholders: the producers, biotechnologists, biologists, and policy makers were in favour of GM palm oil. This can be seen by their high support for GM palm oil (weighted average 5.0 and above) but only moderate encouragement for GM soybean and GM insulin. On the other hand, the biology students were more positive about GM products by showing high support for GM palm oil and GM insulin. The other stakeholders were moderately supportive of the three products surveyed. Analyses of variances were significant for the encouragement of GM soybean (*F* = 3.18, *P* < 0.001), GM palm oil (*F* = 2.59, *P* < 0.01), and GM insulin (*F* = 3.03, *P* < 0.001) across stakeholders. Post hoc tests showed significant difference in the support of the biology students towards all three GM products compared to the Buddhist and Christian scholars, additionally differed with the Islamic scholars with respect to GM soybean and GM insulin, displayed higher rating than the general public in their opinion of GM palm oil, and showed higher rating of GM insulin compared to the Hindu scholars (Table 9). On the other hand, the Christian scholars were found to be the least encouraging of GM soybean and GM palm oil. Post hoc tests confirmed that their level of encouragement for all three GM products as significantly lower than the biology students, additionally lower than the biologist in their rating of GM palm oil, and differed with the producers, politicians, and general public in their level of support for GM soybean (Table 9). The Hindu scholars were the least supportive of GM insulin. Post hoc test showed that their rating was significantly lower than the biology students.

## 4. Discussion

The Klang Valley stakeholders claimed to be not very familiar with GM foods and GM insulin surveyed, with the overall weighted averages below the mid-point value of 4.0 on a scale of 1–7. This finding is not surprising as modern biotechnology has been associated with being “novel” and “complex” with only moderate level of awareness and knowledge among the public, no mandatory labelling of modern biotechnology products in Malaysia and limited coverage of modern biotechnology issues in the Malaysian mass media [52]. Genetically modified palm oil was perceived as slightly more familiar by the Klang Valley stakeholders probably due to its being a local crop. The public in the United Kingdom also had low familiarity with GM foods [46]. The mean score for a familiarity item similarly used in this survey was 1.6 (item how easy to tell whether a food contains the risk) on a scale of 1–5. Using a single question whether the respondents have heard about biotechnology, Gaskell et al. [9] reported that 50% of the Europeans claimed that they were familiar with GM foods but only 27% have heard about pharmacogenetics in 2005. In Philippines, the majority of the stakeholders (85.4%) rated themselves as having only some knowledge of the uses of biotechnology in food production [53]. In Indonesia, the majority (67.7%) of the university students claimed to have only some information about GM foods or organism while only 7.1% believed that they were very well informed [17]. In the same study, Nanere et al. [17] reported that the percentage for the “very well informed and somewhat informed” in Australia are about the same as in Indonesia. Zhang et al. [19] reported that the consumers in urban China have limited knowledge of biotechnology. Eventhough imported GM soybean accounted for the majority of total domestic consumption of soybean, only 18% of the respondents thought that they were consuming GM soybean products. An item asked under the familiarity dimension is “how easy is it for you to know or identify the following food/medicine”. The low score for this item suggests that labelling is needed. Although the main function of labels is to provide information, labelling may also function as a cue for product safety [54, 55] and personal control over the consumption of new food products [10, 31].

This study reveals that acceptance of GM products by the Malaysian stakeholders varies not so much according to the type of applications or products but rather on the intricate relationships between the factors, familiarity, benefit to consumers, and as the moral aspects or the type of gene transfers involved. From this study, it can be seen that one type of GM food (GM palm oil) was the most supported but, on the other hand, the other type of GM food (GM soybean) was the least supported compared to GM insulin (Table 10). When a GM application was perceived as the most familiar, offering major and clear benefit to consumers, and of low moral concern, the risk associated with it would be highly compensated (acceptable) and the application would be strongly encouraged. These patterns were true for the three GM applications surveyed (Table 10). The intricate balancing relationship of the attitudinal factors has been explained by Amin et al. [52, 56]. The findings in this study are supported by some of the earlier studies on public perception towards modern biotechnology and GM foods. Data from the fourth Eurobarometer survey suggested that perceived benefit was found to be a precondition for Europeans' support of biotechnology applications [9, 26, 34] while the moral aspects of GM applications appeared to act as a veto [33, 34].

Overall, the Malaysian stakeholders were the most supportive of GM palm oil (overall weighted average of 4.86) followed by GM insulin (overall weighted average of 4.44) and GM soybean (overall weighted average of 4.29). The weighted averages for overall encouragement in this study are slightly higher than the mean score of Malaysians' attitude towards agricultural biotechnology (52.52 out of total score of 100) in the ISAAA-UIUC (2003a) report. Across Asia, the support for GM foods and medicines was not very encouraging too. Data on public attitude in other Asian countries is rather limited for comparison and those available used different questionnaires. The urban shoppers in Seoul also perceived the benefits of GM foods as moderate too (mean score 3.25 on a scale of 1–5) [20]. In China, 50% of the consumers were willing to accept GM plant products with plant gene source but only 30% of them expressed their acceptance of GM plant products with animal gene [19]. In a more recent survey, Krishna and Qaim [21] reported that the urban consumers in India were slightly more positive with 68% support of Bt vegetables. The level of support towards GM foods and medicine in other Asian countries were lower. Krualee and Napasintuwong [18] reported that about 66% of the Thais consumers in Bangkok were not willing to purchase GM foods. Nanere et al. [17] found that only 19.3% of the Indonesian university students believed GM foods as very safe compared to 32.6% who perceived them as very risky and 38.4% who were unsure. A more comprehensive study conducted by ISAAA-UIUC in 2002 [13–16] reported that although the stakeholders in Asia acknowledged GM crops and insulin as useful, they also believed that those applications posed some risks [14]. The same study found that only 18% of the consumers were supportive of GM crops resistant to pests and diseases compared to 17% consumers who support GM insulin in Thailand. In Indonesia, the support for GM crops was also low with only 24% of the consumers claiming support for GM crops and 25% were supportive of GM insulin [15]. The same pattern could be seen with The Philippine consumers where 20.71% encouraged GM crops and 19.52% supported GM insulin [16]. It is rather interesting to note here that there are similarities in the pattern of support by the Asians. The moral factor is even more important for the Asians in their decision making. Genetically modified insulin which involved the use human gene received lower support compared to GM crops containing plant gene. On the other hand, the Europeans were found to be more encouraging of GM insulin compared to GM crops [12, 34, 45]. Across the globe, the Americans were also found to be more supportive of GM pharmaceuticals compared to pest resistant GM crops [57]. Comparing the encouragement level between countries, the Americans were the most supportive of both GM pharmaceuticals (mean score about 3.3 out of total score of 4.0) and pest resistant GM crop (mean score about 3.0 out of total score of 4.0) [57] than the Europeans and Malaysian stakeholders. The Europeans were also more supportive of GM insulin (mean score of 3.01 out of total score of 4.0) [34, 45] compared to the Malaysian stakeholders but the Malaysian respondents seemed to be slightly more encouraging of herbicide resistant GM soybean (weighted average greater than mid-point value) compared to European support toward insect resistant GM crops in 1999 (mean score about the mid-point value) [34] and in 2002 (less than the mid-point value) [35].

Comparing across stakeholders, the biology students were clearly the most enthusiastic about GM foods and GM insulin surveyed as well as claiming to be the most familiar with those products. This could be because they were still studying and therefore were actively seeking information related to their courses. It is typical of the biology courses in Malaysia to focus on the theory, concepts, and applications/development of GM products rather than on the risk aspects. So it is to be expected that the biology students would be highly enthusiastic about the potentials of GM products. On the other hand, the media subjects were noticeably the most critical of GM foods and GM insulin. This finding again supported the earlier study by ISAAA-UIUC [13], where they reported that the Malaysian journalists showed the highest rating on overall perceived risks of the use of biotechnology in food production. This scenario could be due to the fact that they were more exposed to both the benefits and risks of biotechnology during media coverage. Aerni [28] reported that columnists in the Philippine were in the group critical towards genetic engineering in agriculture while editors were in the ambivalence group. Torres et al. [53] revealed the Philippine's journalists as ambivalent where they acknowledge both the benefits and risks of GM crops.

The NGOs in the Klang Valley region were not strongly against GM foods and GM insulin. Their perception of the risks related to the three GM applications surveyed was in the moderate range, acknowledging moderate benefits and moderately encouraging the three GM applications comparable to the majority of the other stakeholders. This is a favourable finding for the country and in contrast with Aerni's study [28]. Aerni [28] reported that the NGOs in the Philippines were very critical towards genetic engineering in agriculture. The moderate stance of the Malaysian NGOs in the Klang Valley region could be due two factors: firstly, the NGOs in Malaysia are involved in diverse activities ranging from women's movement, Islamic NGOs, environmental NGOs, human rights movement, consumer organizations, and international NGOs branches and, secondly, there are a very limited number of NGOs with a specific interest on modern biotechnology.

Surprisingly and as cause for concern, the biotechnologists and policy makers claimed to have only moderate familiarity with GM foods and GM insulin surveyed and their weighted averages were in the same category (below the mid-point value of 4.0) with the majority of the other stakeholders with the exception of the biology students. Even more worrying is the ignorance of the policy makers who are responsible for making decisions regarding modern biotechnology issues in Malaysia. They professed to have low familiarity with GM soybean and although their familiarity with GM palm oil and GM insulin were in the moderate category, they were within the lowest three ranking together with the religious scholars groups. The reason behind this could be because biotechnologists tended to focus their activities on research and development of new products while the policy makers are mostly biotechnology related academicians or researchers who also share the same priorities.

Attitude of the scientists (biotechnologists and biologists), policy makers, and the producers in the Klang Valley region seemed to be cautious towards GM products. Although their attitudes were inclined towards the positive side compared to the other stakeholders except for the biology students, they also seemed to have some reservations about GM foods and GM insulin. The attitude towards GM products scenario is rather common worldwide. Torres et al. [53] stated that the majority of the scientists, policy makers, and businessmen in the Philippines perceived agricultural biotechnology in food production as beneficial but at the same time almost half of them acknowledged that there are risks associated with GM crops. Aerni [28] also noticed that, in Philippines, the scientists, the government officials, and the politicians were ambivalent in their attitude towards genetic engineering in agriculture. Some of them tended to emphasize the benefits while others highlighted the risks but they had high expectations that genetic engineering can solve the problems confronting the Philippines rice economy. ISAAA-UIUC [13–16] studies also reported that the scientists and policy makers in Asia admitted that they had high levels of personal concerns about biotechnology compared to the other stakeholders but also perceived high levels of benefits related to biotechnology. This might be due to the “novelty” or “complexity” of modern biotechnology. Currently, the focus worldwide is more towards R&D on modern biotechnology products with limited efforts on process and product safety analysis. This proposition is further supported by the finding that the producers, scientists, and policy makers in Malaysia also did not show high confidence in their counterparts.

Among the stakeholders, the Buddhist and Christian scholars were highly concerned about the moral aspects of GM foods and GM insulin surveyed while the Hindu scholars perceived the moral aspects of GM insulin as high. Aerni [28] found that representatives from one group of the churches in the Philippine seemed to be concerned about the ethical aspects of genetic engineering in agriculture. Torres et al. [53] also reported that the religious leaders in Philippines as rating the uses of agricultural biotechnology in food production as the most hazardous compared to other stakeholders. Nelkin [58] also reported that religious groups in USA perceive genetic engineering as an undue intervention into God's creation. On the other hand, the Islamic scholars rated the moral concerns of the three biotechnology products as moderate and their position was comparable to the nonreligious groups. At the same time, the religious scholars also acknowledged the three biotechnology products as having moderate benefits and were moderately supportive of them.

The politicians and the general public were found to have moderate attitude towards GM foods and GM insulin. The consumers in the Philippines were more positive towards GM crops [53] but it should be noted that the general public in this study were more heterogeneous compared to the consumers in Torres's study referred to as the middle-class urban supermarket goers who have had some college education.

## 5. Conclusions

Despite significant developments in modern biotechnology and GM foods worldwide and in Malaysia, the Klang Valley stakeholders' overall attitude towards GM foods and GM insulin was found to be cautious. Although they acknowledged the presence of moderate perceived benefits associated with GM foods and GM insulin surveyed and were moderately encouraging of them, they were also moderately concerned about the risks and moral aspects of the three products as well as moderately accepting the risks. Results from this study revealed that acceptance of modern biotechnology products by the stakeholders varies not so much according to the type of applications or products but rather on the intricate relationships between all the factors, familiarity, benefit to consumers, and the moral aspects or the type of gene transfers involved. If the biotechnology application offers high and clear benefit to consumers and is of low moral concern, the risk associated with it would be highly compensated (acceptable) and the application would be highly encouraged. It is suggested that biotechnologists and industries assess the benefit, risk, and moral aspects of new GM food and GM applications/products to gauge public acceptance of the applications before embarking on R&D and commercialization to avoid the loss of huge amount of financial and labour investments if the products turn out to be unacceptable to consumers. Labelling GM foods and GM products is also recommended to increase consumers' confidence in the products besides the need to make available scientific evidence on the safety of modern biotechnology products by independent researchers.

The research findings serve as a useful database for understanding public acceptance of GM foods in a developing country to understand the social construct of public attitude towards GM foods. A more in-depth study needs to be carried out to evaluate the reasons for the low familiarity of the Malaysian public especially among the biotechnologists and policy makers on GM foods and to explore the cautious attitude of the scientists (biotechnologists and biologists), policy makers, and the producers in the Klang Valley region towards GM foods and products. There is also a need to understand the religious perspectives of various religions on the moral aspects of genetic modification as well as looking at the actual reasons behind the critical nature of the people in media.

## Figures and Tables

**Table 1:** Operational definitions of the stakeholders.

Stakeholders	Definitions
(1) Producers	Management representatives from food, agriculture, pharmaceutical, and agrochemical industry and organizations related to or with potential interest in biotechnology
(2) Biotechnologists	Science and health professionals involved in biotechnology research and development (R&D)
(3) Biologists	Life science and health professionals not involved in biotechnology (R&D)
(4) Policy makers	Government officers and legislators involved in decision making related to biotechnology
(5) Nongovernmental organizations (NGOs)	Leaders of NGOs with an interest in biotechnology
(6) Media	Media writers and editors from major newspapers and broadcasters from major television and radio whose primary beat is science and technology
(7) Politicians	Ministers, senators, and parliamentarians
(8) Islamic scholars	Leaders of Islamic organizations, head of houses of worship, and academicians specializing in Islamic studies.
(9) Buddhist scholars	Leaders of Buddhist organizations, head of houses of worship, and academicians specializing in Buddhist studies.
(10) Christian scholars	Leaders of Christian organizations, head of houses of worship, and academicians specializing in Christianity studies.
(11) Hindu scholars	Leaders of Hindu organizations, head of houses of worship, and academicians specializing in Hindu studies.
(12) Biology students	University and college students majoring in biology
(13) General public	Respondents who does not belong to any of the above categories. They are stratified proportionately according to their occupations classification by Malaysian Standard Classification of Occupations 1998 (MASCO) with a little modification. The managers, senior officials, and legislators were combined with the professional group; the agricultural and fishery workers were combined with the elementary occupation as their percentage was only 0.71% and another category was created for the unemployed.

**Table 2:** Measurement scales, reliability, and validity.

Factor and items	Corrected item-total correlation	*α*	Standardized factor loading	Composite reliability	Average variance extracted (AVE)
Familiarity					
Easy to know	0.595	0.772	0.706	0.775	0.536
Easy judgement	0.653		0.812		
Effect known	0.571		0.671		
Perceived benefit					
Benefit to Malaysian society	0.718	0.868	0.784	0.871	0.578
Enhance product quality	0.746		0.839		
Enhance quality of life	0.772		0.864		
Enhance Malaysian economy	0.668		0.691		
Benefits exceed risks	0.563		0.588		
Perceived risk					
Feeling of anxiety	0.767	0.880	0.855	0.882	0.603
Harm to health	0.814		0.903		
Long-term effect	0.709		0.744		
Catastrophic potential	0.695		0.717		
Overall risk magnitude	0.695		0.633		
Risk acceptance					
Accept if it can boost Malaysian economy	0.681	0.797	0.780	0.797	0.568
Social acceptance	0.625		0.695		
Comparison with other risk	0.624		0.782		
Moral concerns					
Threaten natural order of things	0.568	0.810	0.633	0.818	0.603
“Play god”	0.717		0.844		
Commodity of life	0.705		0.834		
Encouragement					
More rigorous R&D	0.668	0.883	0.715	0.884	0.658
Should be commercialized	0.764		0.840		
Should be given monetary support by government	0.801		0.853		
Overall encouragement	0.748		0.828		

**Table 3:** AVE square roots and correlation matrix of the constructs.

Constructs	Familiarity	Perceived benefit	Perceived risk	Risk acceptance	Moral concerns	Encouragement
Familiarity	**0.732**					
Perceived benefit	0.191***	**0.760**				
Perceived risk	−0.047 (ns)	−0.433***	**0.777**			
Risk acceptance	0.215***	0.625***	−0.498***	**0.753**		
Moral concern	−0.124**	−0.259***	0.315***	−0.305***	**0.776**	
Encouragement	0.208***	0.632***	−0.370***	0.590***	−0.320***	**0.811**

AVE square roots in bold; ***P < 0.001, **P < 0.01.

**Table 4:** Weighted average and post hoc test results for familiarity of GM foods and GM insulin.

Stakeholder	Weighted average ± std dev.*		
	GM soybean	GM palm oil	GM insulin
(1) Producers (*n* = 36)	3.27 ± 1.28	3.40 ± 1.41	3.37 ± 1.38
(2) Biotechnologists (*n* = 30)	2.98 ± 1.20	3.27 ± 1.23	3.17 ± 1.16^12^
(3) Biologists (*n* = 43)	3.01 ± 1.42	3.15 ± 1.42^12^	3.22 ± 1.53^12^
(4) Policy makers (*n* = 40)	2.96 ± 1.32	2.95 ± 1.22^12^	3.02 ± 1.62^12^
(5) NGOs (*n* = 41)	3.38 ± 1.09	3.51 ± 1.17	3.39 ± 1.14^12^
(6) Media (*n* = 38)	3.14 ± 1.28	3.21 ± 1.33	2.97 ± 1.25^12^
(7) Politicians (*n* = 38)	3.10 ± 1.41	3.52 ± 1.36	3.09 ± 1.40^12^
(8) Islamic scholars (*n* = 47)	2.67 ± 0.83	2.85 ± 1.46^12^	2.68 ± 1.38^12^
(9) Buddhist scholars (*n* = 28)	2.60 ± 0.87	2.96 ± 0.83^12^	2.92 ± 0.92^12^
(10) Christian scholars (*n* = 26)	2.11 ± 1.44^12^	2.57 ± 0.97^12^	2.74 ± 1.10^12^
(11) Hindu scholars (*n* = 26)	2.86 ± 1.26	3.08 ± 1.25	2.99 ± 1.65^12^
(12) Biology students (*n* = 46)	3.88 ± 1.26^10^	4.45 ± 1.25^3,4,8,9,10^	4.47 ± 1.56^2,3,4,5,6,7,8,9,10,11,13^
(13) General public (*n* = 578)	3.23 ± 1.29	3.53 ± 1.37	3.28 ± 1.37^12^
Overall (*n* = 1017)	3.14 ± 1.30	3.43 ± 1.36	3.25 ± 1.40

*Post hoc test results showing significant differences at least at *P* < 0.05 between the indicated group and the stakeholders numbered in superscript. Scheffe's test was carried out for GM soybean and GM palm oil while Games-Howell test was carried out for GM insulin.

*Code of stakeholders: ^1^producers, ^2^biotechnologists, ^3^biologists, ^4^policy makers, ^5^NGOs, ^6^Media, ^7^politicians, ^8^Islamic scholars, ^9^Buddhist scholars, ^10^Christian scholars, ^11^Hindu scholars, ^12^biology students, and ^13^general public.

**Table 5:** Weighted average and post hoc test results for perceived benefit of GM foods and GM insulin.

Stakeholder	Weighted average ± std dev.*		
	GM soybean	GM palm oil	GM insulin
(1) Producers (*n* = 36)	4.58 ± 1.55	5.34 ± 1.23^6^	4.93 ± 1.36
(2) Biotechnologists (*n* = 30)	4.24 ± 1.37	4.96 ± 1.05	5.16 ± 1.21^6^
(3) Biologists (*n* = 43)	4.27 ± 1.42	4.93 ± 1.47	4.96 ± 1.48
(4) Policy makers (*n* = 40)	4.28 ± 1.44	5.03 ± 1.13^6^	5.08 ± 1.42
(5) NGOs (*n* = 41)	3.79 ± 1.57^12^	4.51 ± 1.23	4.54 ± 1.42
(6) Media (*n* = 38)	3.73 ± 1.22^12^	4.03 ± 1.40^1,4,12^	4.08 ± 1.33^2,12^
(7) Politicians (*n* = 38)	4.24 ± 1.18	4.91 ± 1.16	4.32 ± 1.43
(8) Islamic scholars (*n* = 47)	3.94 ± 1.47	4.46 ± 1.54	4.23 ± 1.59^12^
(9) Buddhist scholars (*n* = 28)	4.45 ± 0.67	4.84 ± 0.73	4.56 ± 0.77
(10) Christian scholars (*n* = 26)	3.89 ± 1.00^12^	4.40 ± 0.95^12^	4.30 ± 1.13
(11) Hindu scholars (*n* = 26)	3.62 ± 1.80	3.86 ± 1.92	3.57 ± 2.20^12^
(12) Biology students (*n* = 46)	4.82 ± 1.08^5,6,10,13^	5.30 ± 1.05^6,10,13^	5.29 ± 1.32^6,8,11,13^
(13) General public (*n* = 578)	4.16 ± 1.20^12^	4.65 ± 1.18^12^	4.35 ± 1.33^12^
Overall (*n* = 1017)	4.17 ± 1.26	4.69 ± 1.25	4.47 ± 1.40

*Games-Howell post hoc test results showing significant differences at least at *P* < 0.05 between the indicated group and the stakeholders numbered in superscript.

*Code of stakeholders: ^1^producers, ^2^biotechnologists, ^3^biologists, ^4^policy makers, ^5^NGOs, ^6^Media, ^7^politicians, ^8^Islamic scholars, ^9^Buddhist scholars, ^10^Christian scholars, ^11^Hindu scholars, ^12^biology students, and ^13^general public.

**Table 6:** Weighted average and post hoc test results for perceived risks of GM foods and GM insulin.

Stakeholder	Weighted average ± std dev.*		
	GM soybean	GM palm oil	GM insulin
(1) Producers (*n* = 36)	4.44 ± 1.30	4.30 ± 1.28	4.34 ± 1.38
(2) Biotechnologists (*n* = 30)	4.64 ± 1.65	3.92 ± 1.59	3.95 ± 1.75
(3) Biologists (*n* = 43)	4.76 ± 1.37	3.98 ± 1.43	4.06 ± 1.55^6^
(4) Policy makers (*n* = 40)	4.58 ± 1.32	4.18 ± 1.24	4.05 ± 1.58^6^
(5) NGOs (*n* = 41)	4.95 ± 1.07	4.28 ± 1.09	4.63 ± 1.11
(6) Media (*n* = 38)	5.39 ± 1.36	5.08 ± 1.51	5.22 ± 1.38^3,4,12^
(7) Politicians (*n* = 38)	4.70 ± 1.08	4.33 ± 1.22	4.67 ± 1.25
(8) Islamic scholars (*n* = 47)	5.11 ± 1.26	4.19 ± 1.64	4.55 ± 1.50
(9) Buddhist scholars (*n* = 28)	4.53 ± 0.76	4.21 ± 0.94	4.40 ± 0.89
(10) Christian scholars (*n* = 26)	4.71 ± 1.00	4.39 ± 0.81	4.58 ± 0.92
(11) Hindu scholars (*n* = 26)	4.86 ± 1.33	4.47 ± 1.65	4.68 ± 2.04
(12) Biology students (*n* = 46)	4.65 ± 1.29	4.13 ± 1.39	3.95 ± 1.51^6^
(13) General public (*n* = 578)	4.77 ± 1.15	4.41 ± 1.27	4.60 ± 1.26
Overall (*n* = 1017)	4.78 ± 1.20	4.35 ± 1.32	4.52 ± 1.36

*Games-Howell post hoc test results showing significant differences at least at *P* < 0.05 between the indicated group and the stakeholders numbered in superscript.

*Code of stakeholders: ^1^producers, ^2^biotechnologists, ^3^biologists, ^4^policy makers, ^5^NGOs, ^6^Media, ^7^politicians, ^8^Islamic scholars, ^9^Buddhist scholars, ^10^Christian scholars, ^11^Hindu scholars, ^12^biology students, and ^13^general public.

**Table 7:** Weighted average and post hoc test results for risk acceptance of GM foods and GM insulin.

Stakeholder	Weighted average ± std dev.*		
	GM soybean	GM palm oil	GM insulin
(1) Producers (*n* = 36)	4.18 ± 1.20	4.38 ± 1.16	4.20 ± 1.40
(2) Biotechnologists (*n* = 30)	3.94 ± 1.74	4.34 ± 1.63	4.35 ± 1.61
(3) Biologists (*n* = 43)	3.81 ± 1.67	4.21 ± 1.56	4.21 ± 1.66
(4) Policy makers (*n* = 40)	4.06 ± 1.48	4.31 ± 1.35	4.35 ± 1.56
(5) NGOs (*n* = 41)	3.50 ± 1.57	3.98 ± 1.36	3.66 ± 1.39^12^
(6) Media (*n* = 38)	3.13 ± 1.43^9,12^	3.33 ± 1.53^9,12^	3.45 ± 1.67^12^
(7) Politicians (*n* = 38)	3.75 ± 1.29	4.17 ± 1.32	3.87 ± 1.31
(8) Islamic scholars (*n* = 47)	3.76 ± 1.46	4.06 ± 1.51	3.98 ± 1.46
(9) Buddhist scholars (*n* = 28)	4.26 ± 0.70^6^	4.47 ± 0.85^6^	4.15 ± 0.69
(10) Christian scholars (*n* = 26)	3.59 ± 1.15	3.98 ± 1.12	3.81 ± 1.11^12^
(11) Hindu scholars (*n* = 26)	3.43 ± 1.46	3.74 ± 1.59	3.08 ± 1.94^12^
(12) Biology students (*n* = 46)	4.43 ± 1.17^6^	4.70 ± 1.06^6,13^	4.79 ± 1.17^5,6,10,11,13^
(13) General public (*n* = 578)	3.92 ± 1.14	4.13 ± 1.21^12^	4.01 ± 1.21^12^
Overall (*n* = 1017)	3.88 ± 1.28	4.14 ± 1.29	4.02 ± 1.34

*Games-Howell post hoc test results showing significant differences at least at *P* < 0.05 between the indicated group and the stakeholders numbered in superscript.

*Code of stakeholders: ^1^producers, ^2^biotechnologists, ^3^biologists, ^4^policy makers, ^5^NGOs, ^6^Media, ^7^politicians, ^8^Islamic scholars, ^9^Buddhist scholars, ^10^Chrisytian scholars, ^11^Hindu scholars, ^12^biology students, and ^13^general public.

**Table 8:** Weighted average and post hoc test results for moral concerns of GM foods and GM insulin.

Stakeholder	Weighted average ± std dev.*		
	GM soybean	GM palm oil	GM insulin
(1) Producers (*n* = 36)	2.87 ± 1.29^5,6,9,10,11,13^	2.47 ± 1.43^5,6,9,10,11,13^	2.40 ± 1.34^3,5,6,7,8,9,10,11,13^
(2) Biotechnologists (*n* = 30)	3.79 ± 1.79^9,10^	3.52 ± 1.70^9,10^	3.70 ± 1.93^9,10^
(3) Biologists (*n* = 43)	3.73 ± 1.76^9,10^	3.44 ± 1.73^9,10^	3.69 ± 1.71^1,9,10^
(4) Policy makers (*n* = 40)	3.61 ± 1.50^9,10^	3.37 ± 1.51^9,10^	3.49 ± 1.70^9,10^
(5) NGOs (*n* = 41)	4.17 ± 1.85^1,9,10^	3.90 ± 1.59^1,9,10^	3.97 ± 1.68^1,9,10^
(6) Media (*n* = 38)	4.57 ± 1.50^1,12^	4.42 ± 1.33^1,8^	4.58 ± 1.48^1,8,12^
(7) Politicians (*n* = 38)	3.79 ± 1.68^9,10^	3.63 ± 1.53^9,10^	3.76 ± 1.69^1,9,10^
(8) Islamic scholars (*n* = 47)	3.78 ± 1.39^9,10^	3.38 ± 1.31^6,9,10^	3.45 ± 1.36^1,6,9,10^
(9) Buddhist scholars (*n* = 28)	5.59 ± 1.19^1,2,3,4,5,7,8,12,13^	5.00 ± 1.03^1,2,3,4,5,7,8,12,13^	5.26 ± 1.07^1,2,3,4,5,7,8,12,13^
(10) Christian scholars (*n* = 26)	5.74 ± 1.26^1,2,3,4,5,6,7,8,12,13^	5.46 ± 1.36^1,2,3,4,5,7,8,12,13^	5.61 ± 1.14^1,2,3,4,5,7,8,12,13^
(11) Hindu scholars (*n* = 26)	4.72 ± 1.64^1^	4.66 ± 1.81^1^	5.04 ± 2.11^1^
(12) Biology students (*n* = 46)	3.42 ± 1.55^6,9,10^	3.37 ± 1.65^9,10^	3.39 ± 1.61^6,9,10^
(13) General public (*n* = 578)	4.05 ± 1.45^1,9,10^	3.89 ± 1.51^1,9,10^	4.08 ± 1.48^1,9,10^
Overall (*n* = 1017)	4.05 ± 1.57	3.84 ± 1.58	4.01 ± 1.61

*Games-Howell post hoc test results showing significant differences at least at *P* < 0.05 between the indicated group and the stakeholders numbered in superscript.

*Code of stakeholders: ^1^producers, ^2^biotechnologists, ^3^biologists, ^4^policy makers, ^5^NGOs, ^6^Media, ^7^politicians, ^8^Islamic scholars, ^9^Buddhist scholars, ^10^Christian scholars, ^11^Hindu scholars, ^12^biology students, and ^13^general public.

**Table 9:** Weighted average and post hoc test results for encouragement of GM foods and GM insulin.

Stakeholder	Weighted average ± std dev.*		
	GM soybean	GM palm oil	GM insulin
(1) Producers (*n* = 36)	4.66 ± 1.63^10^	5.08 ± 1.71	4.75 ± 1.91
(2) Biotechnologists (*n* = 30)	4.36 ± 1.75	5.00 ± 1.65	4.76 ± 1.60
(3) Biologists (*n* = 43)	4.32 ± 1.58	5.24 ± 1.47^10^	4.55 ± 1.81
(4) Policy makers (*n* = 40)	4.44 ± 1.44	5.12 ± 1.38	4.70 ± 1.72
(5) NGOs (*n* = 41)	4.04 ± 1.60	4.69 ± 1.29	4.24 ± 1.70
(6) Media (*n* = 38)	4.18 ± 1.17	4.76 ± 1.20	4.51 ± 1.46
(7) Politicians (*n* = 38)	4.81 ± 1.41^10^	4.93 ± 1.47	4.57 ± 1.59
(8) Islamic scholars (*n* = 47)	3.79 ± 1.57^12^	4.66 ± 1.41	4.19 ± 1.36^12^
(9) Buddhist scholars (*n* = 28)	3.96 ± 0.79^12^	4.51 ± 0.98^12^	4.19 ± 0.93^12^
(10) Christian scholars (*n* = 26)	3.48 ± 1.00^1,7,12,13^	4.19 ± 1.01^3,12^	4.11 ± 1.06^12^
(11) Hindu scholars (*n* = 26)	4.12 ± 1.38	4.29 ± 1.51	3.49 ± 1.95^12^
(12) Biology students (*n* = 46)	4.91 ± 1.30^8,9,10^	5.47 ± 1.04^9,10,13^	5.31 ± 1.21^8,9,10,11,13^
(13) General public (*n* = 578)	4.29 ± 1.25^10^	4.85 ± 1.29^12^	4.40 ± 1.43^12^
Overall (*n* = 1017)	4.29 ± 1.35	4.86 ± 1.33	4.44 ± 1.50

*Games-Howell Post hoc test results showing significant differences at least at *P* < 0.05 between the indicated group and the stakeholders numbered in superscript.

*Code of stakeholders: ^1^producers, ^2^biotechnologists, ^3^biologists, ^4^policy makers, ^5^NGOs, ^6^Media, ^7^politicians, ^8^Islamic scholars, ^9^Buddhist scholars, ^10^Christian scholars, ^11^Hindu scholars, ^12^biology students, and ^13^general public.

**Table 10:** Ranking of the three modern biotechnology applications.

Attitude dimension	Overall weighted average		
	GM palm oil	GM insulin	GM soybean
Familiarity	3.43^1^	3.25^2^	3.14^3^
Perceived benefit	4.69^1^	4.47^2^	4.17^3^
Perceived risk	4.35^3^	4.52^2^	4.78^1^
Risk acceptance	4.14^1^	4.02^2^	3.88^3^
Moral concern	3.84^3^	4.01^2^	4.05^1^
Encouragement	4.86^1^	4.44^2^	4.29^3^

^1,2,3^Ranking.
